# The effects of tranexamic acid on the histopathology of defect healing in an *in vivo* porcine model after gastric and colonic endoscopic submucosal dissection

**DOI:** 10.3389/fmed.2024.1352967

**Published:** 2024-10-28

**Authors:** Anton Bermont, Shay Matalon, Daniel L. Cohen, Vered Richter, Yariv Siman-Tov, Haim Shirin, Sergei Vosko

**Affiliations:** ^1^Gastroenterology and Liver Diseases Institute, Shamir Medical Center, Zerifin, Israel; ^2^Pre Clinical Department, Shamir Medical Center, Zerifin, Israel

**Keywords:** endoscopic submucosal dissection, endoscopy, tranexamic acid, bleeding, complications

## Abstract

**Introduction:**

There is limited data on the histopathological effects of hemostatic agents (HAs) used during endoscopic submucosal dissection (ESD). We used an *in vivo* porcine model to compare the tissue effects of tranexamic acid (TXA) and adrenaline (epinephrine) compared to controls.

**Methods:**

Standard ESD, using a 2 mm flash-knife, was performed in three pigs. Four resections were performed in the stomach and rectum of each pig. Injectate contained 4% succinylated gelatin solution and indigo carmine, plus either TXA, adrenaline, or neither. Pigs were euthanized after 7 days and evaluated by two blinded pathologists.

**Results:**

Twenty-four defects were analyzed. Within each animal no significant histopathological changes were noted between the defects, but differences were noted between the animals. In the stomachs of the TXA and adrenaline pigs, pathology revealed a clear ulcer in the mucosa/submucosa with abundant granulation tissue, while the muscular layer was unaffected. In the control pig’s stomach, the lesions were deeper, transmurally distributed, with inflammation of the muscular and adventitia layers accompanied by severe inflammation and necrosis. Fewer differences were noted in the rectum.

**Conclusion:**

For ESD, HAs such as TXA and adrenaline may have protective effects on the depth and extent of injury to the underlying tissue.

## Introduction

Endoscopic submucosal dissection (ESD) has become a common minimally invasive therapy for early colonic and gastric neoplasia. One of the more serious adverse events is immediate or delayed bleeding which can occur in up to 11.9% of cases (1, 2). To prevent bleeding, several techniques are used including vessel coagulation and the use of adrenaline (epinephrine) as part of injection gel (1). While adrenaline is the most commonly used hemostatic agent, it may lead to increased postprocedural pain and prolong patient observation after the procedure (3).

Tranexamic acid (TXA) is a synthetic derivative of lysine that exerts antifibrinolytic effects by inhibition of lysine binding sites on plasminogen molecules and therefore stabilizes the fibrin meshwork produced by secondary hemostasis (4). During the past few years, TXA has been “rediscovered” and is now used in several fields of medicine to reduce bleeding and transfusion requirements (5–7).

The effectiveness of systemic TXA in gastrointestinal bleeding is controversial. A large, randomized controlled trial of intravenous TXA found that it is unlikely to confer additional benefits beyond the current standard of care in all patients with severe upper or lower GI bleeding (8).

Topical use of TXA may be more beneficial than systemic use as it may provide a higher drug concentration on the wound surface without causing systemic side effects (9). Moreover, according to a few animal studies, it may have a positive effect on wound healing and may reduce the development of fibrosis (10, 11). In gastroenterology (GI) practice, there is only limited data for the topical use of TXA, and there is no data on how TXA affects histopathological changes in GI tract.

Recently, there has been increased attention to the possible histological effects of injectates used during ESD. For example, a commonly used submucosal lifting agent (Orise) was found to be associated with a foreign body-like granulomatous reaction (12).

Therefore, in this study we primarily aimed to assess the safety and histopathological changes related to the submucosal injection of TXA in ESD specimens. This was performed in an *in vivo* porcine model and compared with submucosal injections containing adrenaline and a control group. The secondary aim of the study was to assess differences in intraprocedural and postprocedural bleeding between the injectate substances and their influence on surgical field visualization.

## Materials and methods

This study was designed as a prospective, blinded, controlled pilot study in live animals. All animal experiments were performed at the animal endoscopy center of Shamir Medical Center after the experimental protocol was approved by the Institutional Animal Care and Use Committee of Shamir Medical Center (Ethics number 54/2021). The study was conducted between January and February 2022.

### Animal pre-procedure preparation

Three female domestic pigs with a mean weight of 48 kg were used. The animals were fasted from solid food for 24 h before ESD but were allowed full access to water. For bowel preparation, each animal received 3 L of polyethylene glycol via orogastric tube 24 h prior to the procedure.

### Endoscopy and ESD

General anesthesia was provided by a veterinarian and performed by using ketamine and isoflurane, nitrous oxide, and O_2_ after endotracheal intubation. Continuous pulse oximetry and electrocardiograph findings were monitored throughout the procedure.

Each animal was assigned a different treatment substance (either adrenaline, TXA, or the control group with no hemostatic agent).

For the first animal (Pig1), the standard 9 mL submucosal injectate comprising succinylated gelatin (Gelofusine; B. Braun, Crissier, Switzerland), 1:100000 adrenaline (epinephrine) and indigo carmine, with 1 mL of saline 0.9% was used.

For the second pig (Pig2), 9 mL submucosal injectate comprising succinylated gelatin and indigo carmine with 1 mL of TXA (100 mg/mL) was used.

For the third pig (Pig3), 10 mL of submucosal injectate comprising succinylated gelatin and indigo carmine.

ESD procedures were performed by two attending advanced endoscopists. The endoscopists were blinded to the randomization of the injection substrates until after all study procedures were performed. The syringes for submucosal injection were prepared outside of the endoscopy room. The same volume (20 mL) of injectate was used for each ESD resection.

All procedures were performed by using a commercially available endoscope (Pentax EC-380LKp). Before resection areas median size 3 cm in diameter each were marked at 4 sites in the body of the stomach and 4 in the rectum, mimicking lesions and were easily accessible by the endoscope. Standard ESD using a 2 mm flush-knife (Fuji Flush Knife BTS, Fujifilm, Tokyo, Japan) was used to resect each lesion. Circumferential incisions with standard dissection were applied for gastric lesions, while tunneling techniques were applied for colorectal lesions. At the end of the procedure, all visible vessels were coagulated. No antacids or proton pump inhibitors were given to the pigs.

### Post-ESD evaluation

For the week following the ESD, daily assessments of general health parameters including monitoring for signs of overt bleeding were performed on the animals. Complete blood counts were taken before the procedure and on day 3 after procedure. Diagnostic gastroscopy and colonoscopy were performed after 1 week of follow up (day 7); afterwards, the animals were euthanized by a veterinarian. The 1 week length of time was chosen as it would ensure that ulcers were still visible at each ESD site, while also falling within the time that post-procedure complications such as bleeding often occur.

Samples from the gastric and rectal ESD sites (two samples for each ESD site) from each of the 3 pigs were harvested and fixed in 4% formaldehyde. The tissues were trimmed, placed in embedding cassettes, and processed routinely for paraffin embedding. A total of 48 cassettes were prepared per animal. Paraffin sections (4 microns thick) were cut, placed on glass slides, and stained with Hematoxylin & Eosin (H&E).

### Outcome parameters and statistics

During the ESD procedures, an assessment of intraprocedural bleeding was performed. Bleeding less than 1 min was defined as minor, lasting more than 1 min with partial interference in visualization or in which coagulation treatment was needed was defined as moderate, and pulsatile bleeding or complete interference with visualization was defined as severe.

The slides were subjected to histopathological evaluation by two expert veterinary pathologists who were blinded to the study groups. Additionally, each pathologist graded the histopathology slides while blinded from the scores given by the other pathologist. The histological slides were examined, described, and scored using a semi-quantitative grading scale. This established 5-point scale graded the severity of the histopathological changes in terms of inflammation, necrosis, and granulation (13).

Grade 1—Minimal inflammation/necrosis/granulation tissue. Findings (<10 inflammatory cells per ×20 magnification, up to 25% necrosis/granulation tissue).

Grade 2—Mild inflammation/necrosis/granulation tissue. Findings (>10 <25 inflammatory cells per ×20 magnification, 25–50% necrosis/granulation tissue).

Grade 3—Moderate inflammation/necrosis/granulation tissue. Findings (>25 <50 inflammatory cells per ×20 magnification, 50–75% necrosis/granulation tissue).

Grade 4—Severe inflammation/necrosis/granulation tissue. Findings (>50 inflammatory cells per ×20 magnification, 75–100% necrosis/granulation tissue).

Finally, a global score (from 0 to 4) was given to the stomach and rectum of each animal as a summary of the severity of the histopathological findings.

In the event of a difference in any of the scores between the two blinded pathologists, the pathologists would discuss the findings until a consensus score was agreed upon.

Given the small number of pigs used, the study was not powered for formal statistical comparisons between the groups.

## Results

### Procedures

Eight ESDs were performed on each animal, 4 in the proximal stomach body and 4 in the rectum. Details of the procedures can be found in Table 1.

**Table 1:** Results of gastric and rectal endoscopic submucosal dissection in the *in vivo* model.

	Pig1 (adrenaline)	Pig2 (TXA)	Pig3 (control)
Total ESD stomach, min	240	275	215
Total ESD rectum, min	130	115	180
Total gastric resection area, cm^2^	38	41	36
Total rectum resection area, cm^2^	31	32	36
Bleeding episodes in stomach, *N*	4	3	3
Bleeding episodes in rectum, *N*	3	0	0
HB before procedure, mg/dL	9.2	10.8	11.9
Hb level on day 3, mg/dL	11.6	12.8	15.3
WBC before, K/mcL	19.1	12.2	16.1
WBC on day 3	24.4	16	21

^*^Hb normal range 10.1–15.1 mg/dL; WBC normal range 6.7–21.8 K/mcL.

### Repeat endoscopies on day 7

Endoscopic examinations were performed after 1 week of follow-up. There was no significant difference in the gross appearance of the wound defects between the groups. All defects had the appearance of clean-based ulcers.

### Histology

**Figure 1:**
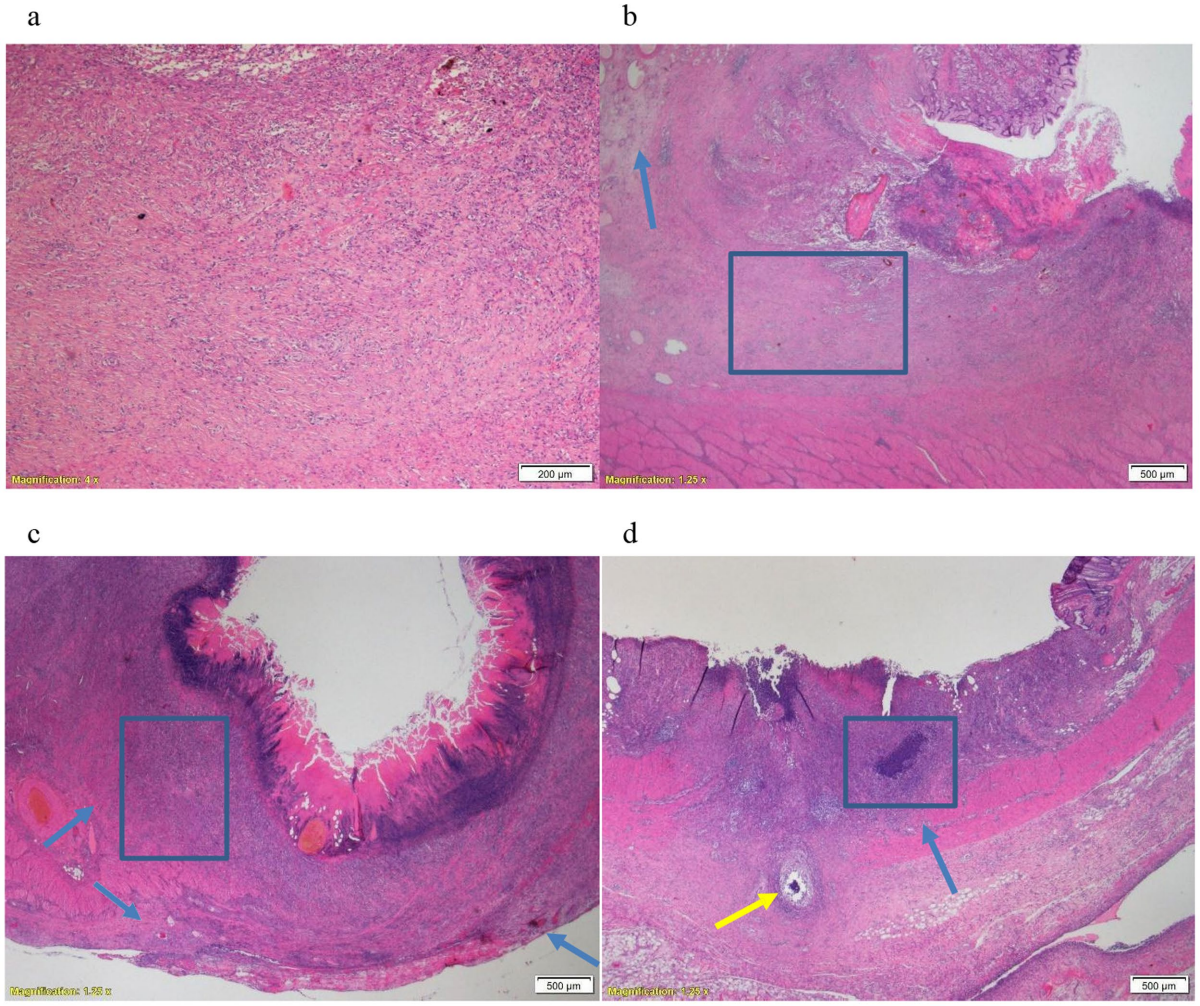
**(a)** Animal Pig2, stomach. Junction of the lesion, abundant amount of granulation tissue and a moderate inflammatory reaction with no apparent necrosis. X4, H&E. **(b)** Animal Pig2, stomach. Junction of the lesion, edema under the intact mucosa (arrow) and an intact muscular layer. X1.25, H&E. **(c)** Animal Pig3, stomach. Very deep lesion, transmural involving the muscular layer and the adventitia with severe inflammation and necrosis (arrows). X1.25, H&E. **(d)** Animal Pig1, colorectum. Deep lesion with severe inflammation and necrosis (blue arrow) and perforation of the muscular layer (yellow arrow). X1.25, H&E.

Within each animal, there were no significant histopathological changes noted between the defects. However, differences were noted in histopathological changes between the animals. In the stomach of first 2 animals (adrenaline and TXA), pathological examination revealed a clear ulcer in the mucosa and submucosa with an abundant amount of granulation tissue and mild inflammation, while the muscular layer was unaffected and intact (Figures 1a,b). There was a marked edema in the junction between the lesion and the intact mucosa and submucosa. In the third animal’s stomach (control group), the lesions were deeper, transmurally distributed, with inflammation of the muscular and adventitia layers accompanied by severe inflammation and necrosis (Figure 1c). Thus, in the stomach, Pig3 had higher inflammation and necrosis scores, and lower granulation score, than the other animals (Table 2).

**Table 2:** A semi-quantitative analysis of the histopathological findings using a scoring scale.

Animal No./Tissue		Inflammation	Necrosis	Granulation tissue
Stomach	Pig1	2	0	4
	Pig2	3	1	4
	Pig3	4	4	1
Rectum	Pig1	4	4	2
	Pig2	3	2	2
	Pig3	3	2	2

^*^Pig1—adrenaline; Pig2—TXA; Pig3—control.

The differences between the lesions in the rectum were less observable and prominent between the animals compared to the stomach lesions. Overall, there was more severe inflammation and necrosis in Pig1 (adrenaline) (Figure 1d).

There was good agreement between the two blinded pathologists in terms of these findings. Of the 18 scores given in Table 2, only two scores differed between the pathologists, and these only differed by 1 grade.

**Figure 2:**
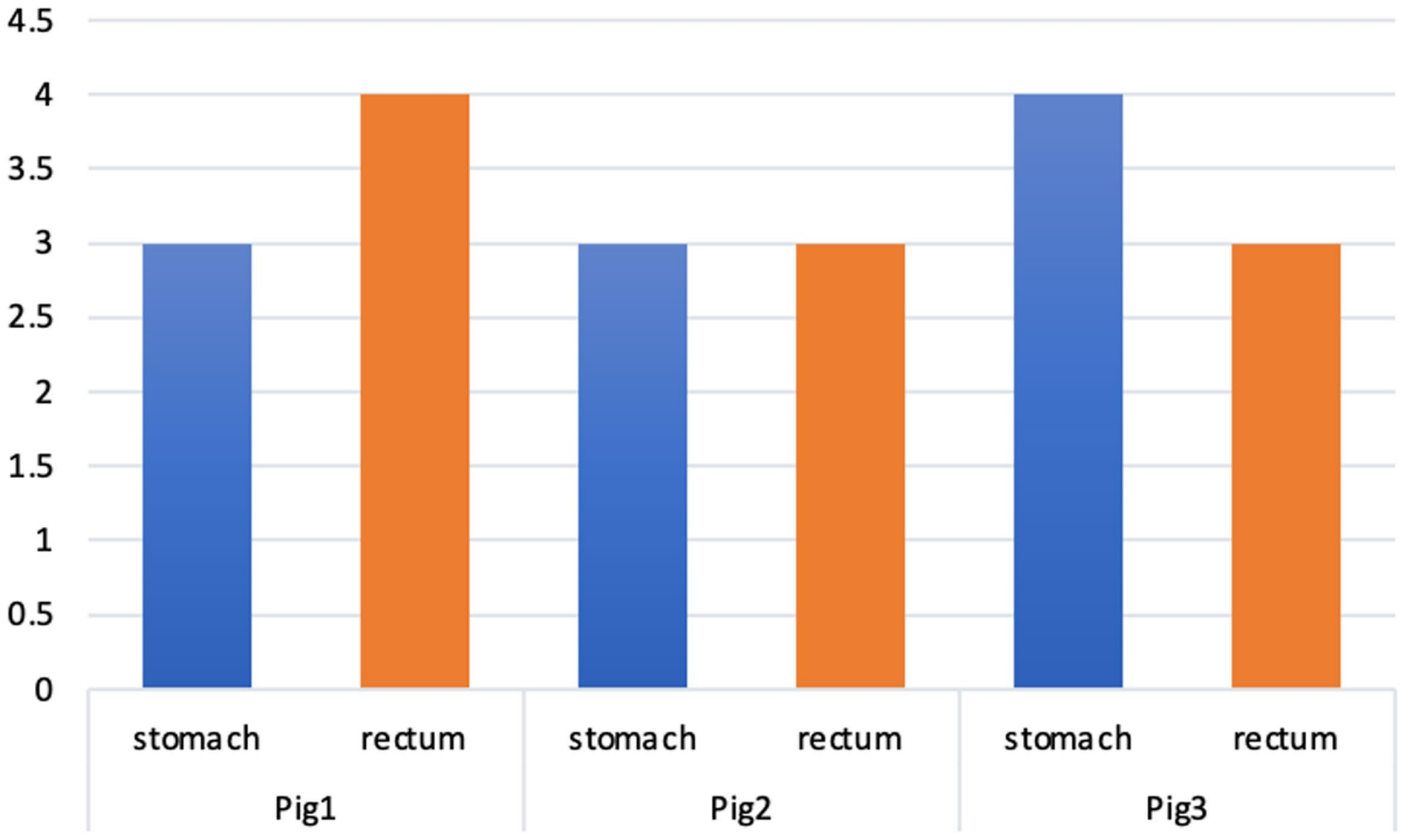
Summary of the histopathological scores in the different animals. ^*^Pig1—adrenalin; Pig2—TXA; Pig3—control.

The summary semi-quantitative histological score is shown in Figure 2. There were no differences between the two pathologists in terms of these scores.

The intact tissue surrounding the lesions appeared normal in all samples. Some edema was noted on the edges of the lesion in some samples. There was no evidence of small vessel thrombosis in any specimens.

### Bleeding

There were no episodes of severe intraprocedural bleeding, but several moderate bleeding episodes occurred (Table 1). There was a similar number between the groups in the stomach, but in the rectum they only occurred in the adrenaline group. All intraprocedural bleeding either stopped spontaneously or with the use of coagulation from the ESD knife.

The coagulation hemostatic grasper was not required. There was no evidence of overt GI bleeding from any of the animals during the 7 days of follow-up, nor was there any difference in general health parameters. Hemoglobin and WBC increased in all animals on day 3 of follow-up.

## Discussion

This is the first study performed in an *in vivo* porcine model to evaluate the histopathologic changes and safety of TXA injected during mucosal resection. In this blinded, randomized, and controlled study, adding a hemostatic agent to the submucosal injectate was associated with less inflammation and necrosis and significant more granulation tissue formation. The positive effects were more prominent in the animals that received TXA and appeared universally in all resection locations. TXA was not associated with significant injury or capillary thrombosis in the tissue adjacent to the resection specimens.

Our findings are consistent with some other studies in animals in which using TXA as a hemostatic agent was associated with an improvement in the healing process. Degirmenci et al. (14) found that topical application of TXA on experimentally induced osteochondral defects in rabbits improved the healing time and was associated with more organized regeneration of subchondral and cartilage tissues. Çevik et al. (15) showed that the topical use of TXA in rats with diaphyseal femoral fractures after fixation exhibited significantly better bone healing than the control group or systemic use of TXA. Additionally, Yuan et al. (16) conducted a study to determine whether TXA could repair the skin barrier by means of tight intercellular junctions using damaged skin models and revealed that TXA can accelerate skin barrier recovery.

It has been proposed that these positive effects on healing may be related to the fibrin-independent effect of TXA on immune function via the plasminogen pathway which is known as an inflammatory regulator that can accelerate wound healing (17–19). For example, it has been shown that administration of TXA in heterozygous Plg^+/−^ mice can restore plasminogen’s effect and improve healing of radiation-induced dermatitis (20). Another recent study evaluated the effects of TXA in patients following cardiac surgery. This study revealed that TXA-mediated plasmin blockade modulates the immune system, reduces injury-induced immunosuppression, and enhanced the expression of immune-activating markers (21).

The beneficial effects of TXA on wound healing may also be related to its beneficial effects on preventing infection. Two studies have shown that TXA reduced the rate of post-operative infections and the need for prolonged antibiotics in patients after orthopedic surgery and cardiac surgery (21, 22).

Epinephrine injection has also been shown to produce positive histopathologic changes during wound healing in animal models and humans. Shirazi et al. (23) performed an experimental hypospadias surgery with topical epinephrine administration as a hemostatic agent. They revealed higher volumes of collagen bundles, and a larger population of fibroblasts were in the histological sections when epinephrine was used which suggested better postoperative structural outcomes. Gacto et al. (24) also showed that subcutaneous administration of adrenaline-lidocaine reduced intraoperative bleeding and accelerated re-epithelialization.

Besides its positive effects on histopathology, our study’s secondary outcomes showed other areas for which TXA injection during ESD appears promising. In our study, the use of TXA was not associated with any significant difference in procedure times, technical difficulties, or surgical field visualization in TXA. Additionally, there was no difference in post-procedural bleeding between groups. These results suggest that TXA is a promising agent and safe for future studies in humans, despite this study being limited by the small number of animals and ESD resections performed.

This study has some limitations. Unfortunately, due to the small number of animals used, formal statistical comparisons between the groups could not be performed, and extrapolating these findings from a small number of animals should be done with caution. Additionally, as with all pathological evaluations, there is an element of subjectivity to reporting the histopathological findings. However, the utilization of an established scoring system used by two blinded pathologists helps mitigate this concern.

In conclusion, we have shown that the topical use of TXA, as well as epinephrine, as an injectate during endoscopic procedures is safe. TXA may also have positive histological effects on post-procedural wound healing.

Future studies assessing its effects during ESD in humans are certainly warranted.

## Data Availability

The raw data supporting the conclusions of this article will be made available by the authors, without undue reservation.
